# Multimodal Active Physiotherapy Versus Multimodal Passive Physiotherapy for Chronic Nonspecific Neck Pain: A Randomized Controlled Trial on Dual Outcomes of Physical and Mental Health

**DOI:** 10.1155/prm/3449647

**Published:** 2025-10-23

**Authors:** Jialin Wang, Shuyi Nie, Ruirui Wang, Yuwei He, Meng Li, Xinwen Cui, Zhoupeng Lu, Hui Zou, Yiping Zhao, Jianfa Xu, Peng Zhao

**Affiliations:** ^1^Sports Rehabilitation Research Center, China Institute of Sport Science, Beijing, China; ^2^School of Sports Medicine and Health, Chengdu Sport University, Chengdu, China; ^3^College of Sports Medicine and Physical Therapy, Beijing Sport University, Beijing, China; ^4^Department of Rehabilitation and Health Care, Anhui College of Traditional Chinese Medicine, Wuhu, China

**Keywords:** chronic nonspecific neck pain, mental health, multimodal active physiotherapy, physical health

## Abstract

**Background:**

Active physiotherapy utilizes patient-centered, sensorimotor-integrated interventions to modulate central nervous system function. This randomized controlled trial (RCT) compared the efficacy of multimodal active physiotherapy (MAP) versus multimodal passive physiotherapy (MPP) in improving physical and mental health in chronic nonspecific neck pain (CNNP) patients.

**Methods:**

This single-blind, stratified parallel-group RCT included 54 patients with CNNP. Participants were randomly assigned to the MAP or MPP group, both receiving 16 treatment sessions (60 min/session) over 8 weeks. Primary outcomes were pain intensity (Numeric Pain Rating Scale) and emotional status (Hospital Anxiety and Depression Scale). Secondary outcomes included patient-perceived change, pain modulation, insomnia symptoms, kinesiophobia, neck function, and mental health–related quality of life (QoL). Intention-to-treat analyses were performed.

**Results:**

Postintervention, there were no significant group-time interactions in the primary outcomes (pain intensity, anxiety, and depression; all *p* > 0.05), despite significant within-group improvements from baseline (all *p* < 0.01). Analyses adjusted for covariates confirmed no between-group differences. For secondary outcomes, the MAP group demonstrated superior improvements compared to the MPP group in conditioned pain modulation, kinesiophobia, and mental health–related QoL; no other significant differences were observed between the groups.

**Conclusion:**

In patients with CNNP, MAP and MPP result in comparable improvements in pain and emotional status; however, MAP demonstrates superior benefits relative to MPP in pain modulation, movement-related fear, and mental health–related QoL.

**Trial Registration:**

Chinese Registry of Clinical Trials: ChiCTR2500104619

## 1. Introduction

Chronic nonspecific neck pain (CNNP), defined as persistent pain lasting beyond 3 months without identifiable pathology, represents a significant clinical and public health burden [1, 2]. This condition, characterized by pain, stiffness, and restricted mobility between the superior nuchal line and the first thoracic vertebra [3], predominantly affects women and sedentary workers aged 45–65 years, with annual incidence rates of 30%–50% [4]. Beyond physical impairment, CNNP is frequently associated with psychological comorbidities, including anxiety, depression, and reduced quality of life (QoL), highlighting the need for holistic treatment approaches [5–7].

Nociceptor inputs can trigger a prolonged but reversible increase in the excitability and synaptic efficacy of neurons in central nociceptive pathways, the phenomenon of central sensitization (CS) [8]. Growing evidence implicates CS as a key pathophysiological mechanism in CNNP [9, 10]. This neuroplastic phenomenon, involving amplified central pain processing, leads to widespread hyperalgesia and impaired endogenous analgesia [8]. Importantly, CS contributes not only to pain persistence but also to the development of pain-related psychological distress [11, 12], suggesting that CS-modulating interventions may offer dual benefits for both physical and mental health outcomes.

Janda's neuromuscular theory proposes that aberrant movement patterns trigger a pathological cascade (muscular imbalance ⟶ maladaptive motor programming ⟶ persistent pain) [12, 13], emphasizing the importance of active patient engagement to restore sensorimotor integration and modulate CS in CNNP [14, 15]. Emerging evidence suggests active treatment strategies (ACSs) may outperform passive treatment strategies (PCSs) by simultaneously enhancing diffuse noxious inhibitory control [15, 16], elevating β-endorphin levels [12], and improving psychological factors such as self-efficacy [17, 18]. Nevertheless, PCSs remain predominant in clinical practice [19–21], despite their limited efficacy in addressing CNNP's biopsychosocial complexity and association with high recurrence rates [22].

Current evidence supports ACSs for managing chronic pain, and the comparative efficacy of active versus passive physiotherapy specifically in CNNP remains unestablished. Based on established theoretical frameworks, active physiotherapy is conceptualized as a treatment model that engages the brain's active participation in an ongoing cycle of sensorimotor integration, facilitated by the patient's participation in guided therapeutic and functional training [23, 24]. In contrast, passive physiotherapy primarily involves therapist-administered manipulations, such as passive joint mobilization and physical agent modalities (PAMs) that rely on traditional biomechanically-driven mechanical input [25].

This randomized controlled trial (RCT) therefore aimed to evaluate whether a multimodal active physiotherapy (MAP) protocol, incorporating Janda's principles and contemporary pain theory [1, 3, 14], demonstrates superior outcomes to multimodal passive physiotherapy (MPP) for both physical and mental health domains in CNNP patients. By comparing the effects of MAP and MPP, this study seeks to propose a novel therapeutic paradigm for CNNP management.

## 2. Methods

### 2.1. Study Design

This was a 8-week, single-blinded, randomized, parallel-group controlled trial. Eligible participants were recruited based on the diagnostic criteria for CNNP established by the American Physical Therapy Association (APTA) [1, 3]. Prior to enrollment, all participants provided written informed consent. The trial was prospectively registered and received ethical approval from the Ethics Committee of the National Institute of Sports Medicine (Code: 20230327-02). The study was conducted in accordance with the ethical principles for human subject research in the Declaration of Helsinki.

### 2.2. Participants

Jialin Wang, Ruirui Wang, and Hui Zou recruited participants aged 18–65 years with neck pain via social media platforms (e.g., WeChat) from May 5 to June 25, 2023. The inclusion criteria were as follows: (1) met APTA criteria for CNNP; (2) baseline average pain intensity score between 3 and 7 on the 11-point Numeric Pain Rating Scale (NPRS), with no shoulder or upper limb symptoms; (3) neck pain occurring at least 3 times per week, with symptoms persisting for 3 months or longer; (4) ability to comprehend and use Chinese fluently and express their intentions clearly; and (5) voluntary participation with signed informed consent.

Exclusion criteria focused on conditions that could contraindicate physical therapy or influence treatment efficacy, including the following: (1) contraindications to physical therapy; (2) neck pain secondary to other systemic/pathological conditions; (3) comorbid psychiatric disorders affecting treatment compliance; (4) history of cervical spine surgery; and (5) concurrent or recent (within 4 weeks prior to enrollment) history of nonpharmacological neck therapy. Participants were permitted to continue their preexisting medications during the study, provided there were no changes to their medication regimen (type, dosage, or frequency). Individuals who had altered their medication regimen within 4 weeks prior to enrollment were excluded. Any initiation, discontinuation, or adjustment of medications during the study was documented as a protocol deviation and resulted in exclusion from the study.

Prior to study enrollment, all participants underwent comprehensive screening through standardized questionnaires, clinical interviews, and physical examinations to assess these conditions.

### 2.3. Randomization, Masking, and Sample Size Calculation

An independent statistician (HYW) conducted computer-generated stratified randomization using STATA 14.0, allocating participants 1 : 1 to MAP or MPP groups, with stratification by sex (male/female) and age (18–45/46–65 years) [26]. Group assignments were concealed within sequentially numbered, opaque sealed envelopes. These envelopes remained unopened until the completion of baseline assessments. Participants remained blinded to treatment allocation throughout the study. Outcome assessors and data analysts were also blinded to group assignment. Treating therapists did not participate in outcome assessment or statistical analysis to maintain blinding integrity, with all evaluations conducted by the same assessor at each time point to ensure consistency.

Sample size estimation was performed a priori using PASS 2021. Based on a two-sample *t*-test with 90% power, a two-sided α of 0.05, and a minimal clinically important difference (MCID) of 2 points (standard deviation [SD] = 2.1) for the outcome (NPRS), a minimum of 23 participants per group was required [24, 27]. Accounting for an anticipated 15% attrition, a total of 54 participants (27 per group) were enrolled.

### 2.4. Procedures

**Figure 1:**
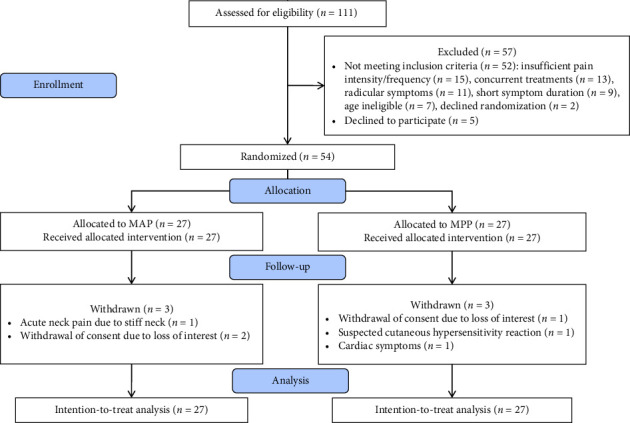
CONSORT flow diagram of the study.

After screening, eligible participants completed baseline assessments after providing written informed consent and were randomized to treatment groups. As per the CONSORT (2010) guidelines [28], the study, including enrollment, allocation, follow-up, and study analysis, is clearly illustrated in the CONSORT flow diagram (Figure 1). Both groups received 16 evidence-based, multimodal progressive therapy sessions, each consisting of 60-min one-on-one interventions delivered by trained physical therapists from July to September 2023. The treatment protocol comprised two sessions per week (with a minimum of 72-h intervals) over eight weeks.

#### 2.4.1. Intervention Measures for the MAP Group

The MAP group received active interventions including pain neuroscience education, active cervical manual therapy, and progressive cervical functional training, all aimed at enhancing self-efficacy and promoting central neuromodulation. The manual therapy encompasses dynamic joint mobilization and myofascial release techniques, delivered with an emphasis on the patient's active movement participation during the procedure [29, 30]. Pain education for the MAP group, to reconstruct patient's cognitive of pain experience [31, 32], included the following: (1) chronic pain causes, plus reasons for its refractoriness and recurrence; (2) psychosocial factors' impacts on pain and function; and (3) importance of active participation for rehabilitation and guidance to encourage independent pain management though sustained exercise.

#### 2.4.2. Intervention Measures for the MPP Group

The MPP group received a treatment regimen consisting of biomechanical education, passive joint mobilization, soft tissue massage, passive static stretching, and PAMs. This approach relied exclusively on therapist-administered techniques and device-based interventions, requiring no active involvement from the patients, who assumed a passive and receptive role throughout the treatment. Patient education for the MPP group included the following: (1) cervical anatomy and biomechanics knowledge, (2) common causes of neck pain, and (3) common self-care methods for neck pain.

All protocols targeted four key domains: pain relief, cervical mobility, muscular strength, and sensorimotor control. Therapists maintained standardized treatment frameworks while allowing individualized progression based on participant response, with session attendance systematically documented and concomitant therapies prohibited. Detailed intervention protocols are presented in Supporting Tables S1 and S2.

### 2.5. Outcomes Measures

Experienced therapists (Shuyi Nie, Meng Li, and Zhoupeng Lu) conducted measurements at the baseline (before the intervention) and after 8 weeks of intervention (end of the intervention). The primary outcome measures were pain intensity and emotional status. Secondary outcomes included patient-perceived change, pain modulation, insomnia symptoms, kinesiophobia, neck function, and mental health–related QoL.

#### 2.5.1. Primary Outcomes

##### 2.5.1.1. NPRS

Mean neck pain intensity was evaluated using an 11-point NPRS, where higher scores indicated greater pain severity, with a MCID of 2 points [33]. The scale demonstrated good feasibility, test–retest reliability, and construct validity when used for evaluating average pain intensity in chronic pain populations [34, 35].

##### 2.5.1.2. Hospital Anxiety and Depression Scale (HADS)

The HADS is a validated 14-item instrument comprising two 7-item subscales for anxiety (HADS-A) and depression (HADS-D), with each subscale scoring from 0 to 21 (total range: 0–42) [36]. Higher scores indicate greater psychological distress. The HADS has been widely used for psychological screening in patients with musculoskeletal disorders [37].

#### 2.5.2. Secondary Outcomes

##### 2.5.2.1. Global Rating of Change (GROC)

The 15-point GROC Scale was used to quantify patient-perceived change, with scores ranging from −7 (*much worse*) to +7 (*much better*) [38]. An MCID was defined as a more than three-point improvement from baseline, as this threshold has demonstrated strong correlation with improvements in pain intensity, functional status, and QoL measures in musculoskeletal populations.

##### 2.5.2.2. Conditioned Pain Modulation (CPM)

The CPM effect was assessed using a cold pressor test as the conditioning stimulus and pressure pain threshold (PPT) as the test stimulus [39]. The nondominant hand of each participant was immersed in room-temperature water (22 ± 1°C) for one minute and then transferred to cold water (12 ± 1°C) for an additional minute, with the hand position maintained throughout. PPT was measured at the upper trapezius (midpoint between C7 and acromion) 30 s after cold exposure, with three repeated trials averaged. CPM effect was calculated as the difference between pre- and posttest PPT values (negative values = impaired CPM; zero/positive values = intact CPM) [40]. Ambient conditions (temperature, humidity, and lighting) were controlled.

##### 2.5.2.3. Insomnia Severity Index (ISI)

The ISI is a seven-item self-reported measure assessing both night-time and day-time symptoms of insomnia, with scores ranging from 0 to 28; higher scores indicate more severe symptoms [41]. The internal consistency of the measure is high (*α* > 0.9) in both clinical and community samples [42].

##### 2.5.2.4. Tampa Scale of Kinesiophobia (TSK)

The TSK is a validated 17-item self-reported questionnaire designed to assess fear of injury based on fear-avoidance behavior and fear of activity. Scores range from 17 (*no kinesiophobia*) to 68 (*severe kinesiophobia*), with higher scores indicating stronger fear-avoidance beliefs [43]. Previous studies have demonstrated excellent test–retest reliability of the TSK in populations with neck pain [44].

##### 2.5.2.5. Neck Disability Index (NDI)

The NDI is a 10-item self-report scale evaluating neck pain and related dysfunction. Each item scores from 0 to 5, with a maximum score of 50. The NDI has demonstrated good reliability, responsiveness, and favorable content and construct validity in individuals with CNNP [45–47].

##### 2.5.2.6. Mental Health QoL (MHQoL)

The MHQoL is a self-report scale designed to assess health-related QoL in people with mental health problems, including both subclinical and clinical populations, as well as users of mental health services [48, 49]. It consists of two components and seven questions on seven dimensions, each with four options. The scale ranges from 0 to 21 points, with higher scores indicating better QoL.

### 2.6. Statistical Analysis

Analyses were performed using SPSS 27. Continuous variables are presented as mean ± SD (normally distributed) or median (interquartile range, non-normally distributed), and categorical variables as frequencies (%). Between-group comparisons used independent sample *t*-tests or Mann–Whitney *U* tests (continuous) and *χ*^2^ tests (categorical). Primary outcomes were analyzed by repeated-measures ANCOVA with adjustment for prespecified covariates (age, sex, body mass index [BMI], total sleep time [TST], sedentary desk time, regular physical activity, and medication use) [26, 50–53]. Secondary analyses employed appropriate between-group (independent *t*-tests or Mann–Whitney *U* test) and within-group (paired *t*-tests or Wilcoxon test) comparisons. Intention-to-treat analysis incorporated all randomized participants with last observation carried forward for missing data. Participants who withdrew were contacted to ascertain reasons for dropping out and encourage continued participation to minimize the loss of data.

## 3. Results

### 3.1. Participants' Characteristics

Between May and June 2023, we screened 111 potential participants, of whom, 54 met eligibility criteria and were randomized to MAP (*n* = 27) or MPP (*n* = 27) groups. Main exclusion reasons included the following: insufficient pain intensity/frequency (28.8%), concurrent nonpharmacological treatments (25.0%), radicular symptoms (21.2%), and short symptom duration (17.3%). By trial completion in September 2023, 48 participants (88.9%) had finished the eight-week intervention. The mean (SD) age of the participants was 39.04 (8.49) years, and 35.19% were male. No statistically significant differences were observed between groups in terms of demographic variables, lifestyle factors, or baseline clinical outcomes (all *p* > 0.05). Detailed baseline characteristics are presented in Table 1.

### 3.2. Treatment Effects on Primary Outcomes

Over time, scores on the NPRS, HADS-A, and HADS-D improved significantly in both groups (time effect, all *p* < 0.01), showing that MAP and MPP interventions reduced pain and improved emotional status. No significant between-group differences were observed in NPRS (*p* = 0.83), HADS-A (*p* = 0.60), or HADS-D (*p* = 0.57). In addition, no significant group-time interaction effects were seen for these outcomes (all *p* > 0.05) (Table 2). However, after adjusting for covariates, no significant effects were noted in terms of time, group, or interaction (all *p* > 0.05). Covariate analysis indicated that age, sex, BMI, TST, sedentary desk time, regular physical activity, and medication use had no significant influence on treatment-related changes in primary outcomes, except for TST, which exerted a significant effect on anxiety and depression (all *p* < 0.01) (Table 3).

### 3.3. Treatment Effects on Secondary Outcomes

Patient-perceived change, as measured by the GROC Scale at postintervention, demonstrated statistically significant gains in both treatment groups (MAP: 12.13 ± 1.48, MPP: 11.79 ± 1.52, both exceeding the MCID of three points). No statistically significant differences were observed between groups (0.33, 95% CI: −0.48 to 1.15, Cohen's *d* = 0.22, *p*=0.42) (Table 4).

Postintervention analysis revealed significant within-group improvements for both protocols. The MAP group demonstrated statistically significant improvements in CPM effect, insomnia severity, kinesiophobia, neck function, and mental health–related QoL, while the MPP group showed significant differences in insomnia severity, neck function, and mental health–related QoL. Comparative analyses demonstrated superior improvements in the MAP group compared to the MPP group across the three outcomes: CPM effect (0.75, 95% CI: 0.36 to 1.14, Cohen's *d* = 0.59, *p* < 0.01), kinesiophobia (6.70, 95% CI: 1.46 to 11.95, Cohen's *d* = −1.48, *p*=0.01), and mental health–related QoL (1.67, 95% CI 0.07 to 3.26, Cohen's *d* = −0.96, *p*=0.04) (Table 4).

### 3.4. Incidence of Adverse Events

There was no significant difference in the incidence of adverse events between groups (2.08, 99% CI: 0.18 to 24.41, *p*=0.55), with 1 withdrawal in the MAP group due to acute neck pain and 2 withdrawals in the MPP group due to cutaneous hypersensitivity and cardiac discomfort (Table 5).

## 4. Discussion

In this RCT comparing MAP and MPP for CNNP, both interventions yielded significant improvements in the primary outcomes of pain intensity and emotional status. Analysis of secondary outcomes, however, revealed distinct therapeutic profiles between the two approaches. While MAP and MPP demonstrated comparable efficacy in patient-perceived treatment benefits, insomnia symptoms alleviation, and neck functional recovery, MAP produced superior improvements in CPM effect, kinesiophobia reduction, and mental health–related QoL. These findings suggest that MAP may offer specific benefits in modulating neurophysiological mechanisms and addressing fear-avoidance behaviors. The observed improvements in CPM effect and kinesiophobia may contribute to lower pain recurrence and lead to sustained clinical benefits over time, an important aspect that warrants further investigation through long-term follow-up, which was not available in the present study.

### 4.1. Analysis of Physical Health–Related Outcomes

Our study found comparable improvements in pain intensity and neck disability between MAP and MPP groups, aligning with evidence that both interventions alleviate symptoms [22, 54, 55]. This may reflect distinct mechanisms: MPP's rapid anti-inflammatory and analgesic effects [56] versus MAP's delayed but potentially superior central actions [57, 58]. Notably, MAP showed enhanced CPM than the MPP group, suggesting stronger activation of endogenous pain inhibitory pathways [59]. The amelioration of pain sensitization through active exercise may be linked to alterations in pain processing and patient-reported outcomes [60]. Neuroimaging evidence reveals that chronic pain induces structural reorganization and hyperactivation of the anterior cingulate cortex, while lactate metabolism has been implicated in pain chronification [61, 62]. These findings collectively suggest substantial interindividual variability in exercise-induced hypoalgesia among chronic pain patients [63]. Consequently, further research is warranted to validate the clinical predictive value of CPM assessments [64].

### 4.2. Analysis of Mental Health–Related Outcomes

The results demonstrated that the MAP group showed significantly greater improvement in kinesiophobia compared to the MPP group, supporting the established association between kinesiophobia and maladaptive pain cognitions [65]. This superiority may stem from the following: (1) targeted pain neuroscience education, elucidating the analgesic mechanisms of active movement [66], and (2) its holistic intervention proving more effective than MPP's biomechanical mechanism in establishing appropriate chronic pain cognitions [67]. These findings suggest that behavioral interventions integrating cognitive education may be superior to purely biomechanical approaches for improving kinesiophobia.

Research has confirmed a well-documented bidirectional relationship between chronic pain and psychological/sleep disturbances [5, 68, 69], wherein chronic pain both exacerbates and is exacerbated by anxiety, depression, and sleep disorders. Current evidence indicates that physiotherapy has limited efficacy in improving anxiety and depression symptoms [70], with cognitive behavioral therapy remaining the preferred intervention for psychological improvement [71, 72]. The potential psychological benefits of physical therapy may primarily result from indirect effects of symptom relief. In this study, the MAP protocol failed to demonstrate significant psychological benefits, potentially due to its lower exercise intensity being insufficient to adequately activate antidepression mechanisms [73, 74]. Furthermore, chronic pain may adversely affect psychological status through sleep disruption [75], which aligns with the observed consistency between sleep assessments and psychological indicators in this study.

### 4.3. Global Perceived Change and MHQoL

Patient's global perception improvement is influenced by multiple factors, particularly rehabilitation beliefs [76]. QoL is modulated by biopsychosocial factors comprising pain catastrophizing cognition [77], activity limitation [78], and insufficient social support [79, 80], which contribute synergistically to functional impairment and psychological distress [81]. Notably, the MAP group's treatment protocol, combining pain neuroscience education with active exercise guidance, addressed this situation through targeted interventions: by scientifically explaining pain mechanisms to establish accurate cognitions and reduce kinesiophobia, alleviating chronic pain–related negative emotions [82], and providing sustained social support via professional therapeutic accompaniment and health education. This comprehensive intervention model achieves synergistic effects across cognitive, behavioral, and social support dimensions, thereby facilitating holistic improvement.

### 4.4. Limitations

This study has several limitations. First, a key limitation of this study is the absence of follow-up assessments, which constrains our ability to evaluate the long-term sustainability of observed improvements. Second, the predominantly young study population with mild pain symptoms may limit the generalizability of findings to the broader CNNP population. Third, the absence of a no-treatment/placebo control group means the observed treatment effects could be influenced by natural disease progression and placebo effects. Finally, the conclusions are specific to the multimodal intervention protocol evaluated in this study of CNNP patients, and its applicability to other chronic musculoskeletal pain conditions requires further investigation.

## 5. Conclusion

Both MAP and MPP interventions demonstrated comparable effectiveness in reducing pain and improving functional outcomes in patients with CNNP. However, the MAP showed superior benefits in both mental health outcomes and overall status. Future research should extended follow-up periods to better evaluate long-term therapeutic outcomes, including recurrence rates and psychological improvements, while employing more objective neuroimaging assessments such as functional magnetic resonance imaging to investigate CS mechanisms in chronic musculoskeletal disorders.

## Figures and Tables

**Table 1:** Baseline characteristics.

Characteristic	MAP (*n* = 27)	MPP (*n* = 27)	*p* value
Demographic			
Age (years), mean ± SD	40.04 ± 9.05	38.04 ± 7.93	0.39
Sex (men), *n* (%)	10 (37.0%)	9 (33.3%)	0.78
Dominant hand, right (%)	27 (100%)	26 (96.30%)	0.31
BMI (kg/m^2^), mean ± SD	22.38 ± 2.48	23.00 ± 2.79	0.39
Education level, *n* (%)			> 0.05^a^
Postsecondary education	27 (100%)	27 (100%)	
Employment status, *n* (%)			0.85
Part-time employed	1 (3.70%)	1 (3.70%)	
Full-time employed	22 (81.48%)	23 (85.19%)	
Full-time student	3 (11.11%)	1 (3.7%)	
Retired	1 (3.70%)	2 (74.07%)	
Lifestyle, *n* (%)			
TST			0.48^a^
> 8 h	2 (7.41%)	3 (11.11%)	
6–8 h	24 (88.89%)	21 (77.78%)	
< 6 h	1 (3.70%)	3 (11.11%)	
Sedentary desk time			0.48^a^
> 8 h	13 (48.15%)	11 (40.74%)	
5–8 h	6 (22.22%)	8 (36.36%)	
2–5 h	7 (25.93%)	4 (14.81%)	
< 2 h	1 (3.70%)	4 (14.81%)	
None	1 (3.7%)	2 (7.41%)	
1–3 per night	26 (96.30%)	25 (92.59%)	
Regular physical activity, *n* (%)	13 (52.00%)	15 (55.56%)	0.59
Medication use			0.83
No medication use	9 (33.33%)	8 (29.63%)	
Topical medication	16 (59.26%)	18 (66.67%)	
Systemic medication	2 (7.41%)	1 (3.70%)	
Etiology of the initial neck pain episode			> 0.05
Exercise-related/biomechanical	1 (3.70%)	2 (7.41%)	
Lifestyle-related	26 (96.30%)	25 (92.59%)	
Outcomes at baseline, mean ± SD			
Pain (NPRS)	4.59 ± 1.15	4.44 ± 1.25	0.77
Anxiety (HADS-A)	6.33 ± 3.46	5.96 ± 3.14	0.81
Depression (HADS-D)	3.85 ± 3.12	4.15 ± 2.18	0.84
Patient-perceived efficacy (GROC)	NA	NA	NA
CPM effect (PPT) (kg/cm^2^)	1.14 ± 1.04	1.26 ± 0.79	0.79
Insomnia symptoms (ISI)	10.19 ± 6.10	8.11 ± 5.12	0.43
Kinesiophobia (TSK)	38.74 ± 6.04	40.22 ± 11.94	0.79
Neck function (NDI)	12.59 ± 4.99	12.22 ± 4.17	0.77
Quality of life (MHQoL)	25.09 ± 3.64	25.70 ± 3.07	0.69

*Note:* BMI = body mass index; GROC = global rating of change; HADS = Hospital Anxiety and Depression Scale; ISI = Insomnia Severity Index; MAP = multimodal active physiotherapy; MPP = multimodal passive physiotherapy; *n* = number of observations; NA = not available; NDI = Neck Disability Index; NPRS = Numeric Pain Rating Scale; PPT = pressure pain threshold; SD = standard deviation; TSK = Tampa Scale of Kinesiophobia; TST = total sleep time; MHQoL = mental health quality of life.

^a^Mann–Whitney *U* test.

**Table 2:** Effects of MAP vs. MPP on primary outcomes.

Assessment^a^ (8 weeks)	Mean ± SD		Mean difference (95% CI)	*p* value (group)		*p* value (time)		*p* value (interaction)	
	MAP (*n* = 27)	MPP (*n* = 27)		Unadjusted	Adjusted	Unadjusted	Adjusted	Unadjusted	Adjusted
Pain, NPRS	2.59 ± 1.15^∗∗^	2.85 ± 0.99^∗∗^	−0.41 (−1.09 to 0.27)	0.83	0.93	**< 0.01**	0.10	0.23	0.19
Anxiety, HADS-A	5.04 ± 3.57^∗^	4.56 ± 2.83^∗∗^	0.11 (−1.31–1.53)	0.60	0.93	**< 0.01**	0.64	0.88	0.49
Depression, HADS-D	2.74 ± 2.03^∗^	3.11 ± 2.06^∗∗^	−0.07 (−1.24 to 1.09)	0.57	0.28	**< 0.01**	0.19	0.90	0.95

*Note:* CI = confidence interval; HADS = Hospital Anxiety and Depression Scale; MAP = multimodal active physiotherapy; MPP = multimodal passive physiotherapy; *n* = number of observations; NPRS = Numeric Pain Rating Scale; SD = standard deviation.

^a^Outcome assessment scales are explained in the Measurements subsection of the Methods Section.

^∗^ *p* < 0.05, ^∗∗^*p* < 0.01 (within-group comparisons).

**Table 3:** Results from covariate analysis.

Covariates	*p* value		*p* value		*p* value	
	Time	Pain	Time	Anxiety	Time	Depression
Age	0.06	0.32	0.47	0.31	0.22	0.38
Sex	0.84	0.64	0.22	0.10	0.81	0.35
BMI	0.12	0.83	0.11	0.06	0.10	0.17
TST	0.46	0.53	0.93	**< 0.01**	0.71	**< 0.01**
Sedentary desk time	0.37	0.92	0.70	0.68	0.62	0.86
Regular physical activity	0.30	0.17	0.15	0.76	0.18	0.35
Medication use	0.28	0.95	0.15	0.28	0.45	0.80

*Note:* BMI = Body Mass Index; Pain/Anxiety/Depression = the effect of covariates on the pain/anxiety/depression; Time = interaction effect between covariates and time; TST = total sleep time.

**Table 4:** Effects of MAP vs. MPP on secondary outcomes.

Assessment^a^ (8 weeks)	Mean ± SD		Mean difference (95% CI)	Cohen's *d*	*p* value
	MAP (*n* = 27)	MPP (*n* = 27)			
Patient-perceived change (GROC)	12.13 ± 1.48^∗∗^	11.79 ± 1.52^∗∗^	0.33 (−0.48–1.15)	0.22	0.42
CPM effect (PPT)	1.61 ± 1.25^∗∗^	0.98 ± 0.86	0.75 (0.36–1.14)	0.59	**< 0.01**
Insomnia symptoms (ISI)	6.89 ± 4.24^∗^	6.00 ± 3.65^∗∗^	−0.78 (−3.82 to 2.27)	0.22	0.61
Kinesiophobia (TSK)	29.11 ± 7.10^∗^	37.29 ± 2.30	6.70 (1.46–11.95)	−1.48	**0.01**
Neck function (NDI)	7.00 ± 3.33^∗∗^	7.74 ± 3.56^∗∗^	−1.11 (−3.41 to 1.19)	−0.26	0.34
Quality of life (MHQoL)	20.81 ± 2.38^∗∗^	23.09 ± 2.38^∗∗^	1.67 (0.07–3.26)	−0.96	**0.04**

*Note:* CI = confidence interval; CPM = conditioned pain modulation; GROC = global rating of change; ISI = Insomnia Severity Index; MAP = multimodal active physiotherapy; MHQoL = mental health quality of life; MPP = multimodal passive physiotherapy; NDI = Neck Disability Index; PPT = pressure pain threshold; SD = standard deviation; TSK = Tampa Scale of Kinesiophobia.

^a^Outcome assessment scales are explained in the Measurements subsection of the Methods Section.

^∗^ *p* < 0.05 and ^∗∗^*p* < 0.01 (within-group comparisons).

**Table 5:** Differences in adverse events between MAP and MPP.

Group	Adverse events (%)	Fisher's exact test (*p*)	RR (99% CI)
MAP	1 (3.70%)	0.55	2.08 (0.18–24.41)
MPP	2 (7.40%)		

*Note:* CI = confidence interval; MAP = multimodal active physiotherapy; MPP = multimodal passive physiotherapy; RR = relative risk.

## Data Availability

The data and materials supporting the findings of this study are available upon request. Researchers interested in accessing the data should contact Jialin Wang (jialinlynne@outlook.com).

## Nomenclature

ACSs: Active treatment strategies
APTA: American Physical Therapy Association
CNNP: Chronic nonspecific neck pain
CPM: Conditioned pain modulation
CS: Central sensitization
GROC: Global rating of change
HADS: Hospital Anxiety and Depression Scale
ISI: Insomnia Severity Index
MAP: Multimodal active physiotherapy
MCID: Minimal clinically important difference
MHQoL: Mental health QoL
MPP: Multimodal passive physiotherapy
NDI: Neck Disability Index
NPRS: Numeric Pain Rating Scale
PAMs: Physical agent modalities
PCSs: Passive treatment strategies
PPT: Pressure pain threshold
QoL: Quality of life
RCT: Randomized controlled trial
SD: Standard deviation
TSK: Tampa Scale of Kinesiophobia

## Supporting Information

Supporting Table 1: MAP protocols in detail.

Supporting Table 2: MPP protocols in detail.
