# Supplementary material for: Multimodal Active Physiotherapy Versus Multimodal Passive Physiotherapy for Chronic Nonspecific Neck Pain: A Randomized Controlled Trial on Dual Outcomes of Physical and Mental Health
Source: Pain Res Manag. 2025 Oct 23;2025:3449647. doi: 10.1155/prm/3449647 (PMC12575025; doi:10.1155/prm/3449647)
Supplement: Supporting Information — Additional supporting information can be found online in the Supporting Information section. [file 3449647.f1.docx]

Supplementary Table 1. Multimodal Active Physiotherapy (MAP) protocols in details

| **Phase** | **Objectives** | **Interventions** | **Duration** |
| --- | --- | --- | --- |
| **Phase 1**  (Weeks 1-3, 6 sessions, 60 minutes per session) | 1. Relieve neck pain 2. Cervical mobility improvement | 1. **patient education**  - video-based pain neuroscience education - illustrated handbook review - therapist-guided discussion   Note: education occurs during the first session only; patients retain the handbook for continued learning. | 20 minutes |
|  |  | 1. **Mobilization with movement** (Mulligan concept)  - C3-C7 SNAG (for flexion/extension/rotation/lateral flexion restrictions) - C5-C7 lateral SNAG (for extension/lateral flexion/rotation restrictions) | Initial session: 10 minutes; subsenquent sessions: 20 minutes |
|  |  | 1. **Myofascial release techniques**  - Suboccipital muscles - Sternocleidomastoid - Upper trapezius - Levator scapulae - Anterior/middle scalenes - Splenius capitis - Rhomboids - Serratus anterior - Pectoralis major/minor | 2-3 minutes per muscle |
|  |  | 1. **Trigger Point Pressure Release** (with fascial ball)  - Trapezius (upper/middle fibers) - Levator scapulae - Pectoralis major/minor   Note: Trigger point pressure release was applied to the aforementioned muscles while patients performed active stretching of the corresponding muscle groups (2-3 minutes per target area) | Initial session: 10 minutes; subsenquent sessions: 20 minutes |
| **Phase 2**  (Weeks 4-6, 6 sessions, 60 minutes per session) | 1. Improve thoracic spine mobility 2. Enhance cervical stability and motor control | 1. **Dynamic Joint Mobilization Techniques** (Mulligan)  - Thoracic SNAG (Progressive Spinal Articulation for Anterior, Posterior, and Lateral Flexion) - Costovertebral MWM (Improve Costovertebral Lateral Flexion Pain) | 10 minutes |
|  |  | 1. **Thoracic Mobility Training**  - Quasi-Breathing Exercises - Book-Turning Exercises - Thoracic Rotational Mobilization | 10 minutes |
|  |  | 1. **Advanced Cervical Stability and Motor Control Training** (Progressive Training on Stable and Unstable Surfaces)  - Deep Cervical Muscle Sensing and Strength Training - Cervical Neurological Developmental Stage Support Training (Prone, Supine, Quadruped, and Standing Positions) Combined with Upper Limb Movements - Cervical Directional Elastic Band Resistance Training - Cervical Movement Equilibrium Training | 25 minutes |
|  |  | 1. **Advanced Scapulohumeral Stability Training** (Progressive Training on Stable and Unstable Surfaces)  - Standing T-Shape Training: Middle Trapezius Muscles - Standing Y-Shape Training: Inferior Trapezius Muscles - Standing W-Shape Training: Superior Trapezius Muscles - Scapular Push-Up Training | 15 minutes |
| **Phase 3**  (Weeks 7-8, 4 sessions, 60 minutes per session) | Proprioception enhancement | **Sensorimotor Training** (Stable to Unstable surfaces progression)   - Cervical joint position sense training - Cervical kinesthesia training - Oculomotor control exercises: Smooth pursuit eye movements; Gaze stabilization training; Eye-head coordination drills | 60 minutes |

MAP protocol Note: Treatment progression and advancement were appropriately adjusted based on individual patient conditions to achieve optimal exercise intensity. Standardized patient education was conducted only during the initial 20-minute treatment session. Participants in the active intervention group performed approximately 5 minutes of diaphragmatic breathing adjustment prior to each session. All techniques and exercises were progressively selected based on treatment progression and individual patient capability, with functional training strictly performed within pain-free parameters.

Supplementary Table 2. Multimodal Passive Physiotherapy (MPP) protocols in details

| Phase | Objectives | Interventions | Duration |
| --- | --- | --- | --- |
| Phase 1  (Weeks 1-3, 6 sessions, 60 minutes per session) | 1. Relieve neck pain 2. Improve cervical mobility | 1. Patient Education  - View cervical anatomy educational videos - Read educational brochures - Discuss with therapist   Note: Patient education is only conducted during the first treatment session; patients are encouraged to continue learning with the provided brochures after treatment completion | 20 minutes |
|  |  | 1. Physical Therapy  - Ultrasound Therapy - Compex Neuromuscular Stimulation - Shockwave Therapy (2 sessions every 3 weeks, up to 1 session per week)   Note: Focus on cervical and shoulder pain points, avoiding major blood vessels and nerves | 25 minutes |
|  |  | 1. Joint Mobilization Techniques (Maitland)  - C3-7 Cervical Central PA Mobilization (Improve Extension Mobility) - C3-7 Cervical Lateral PA Mobilization (Improve Lateral Rotation) - Cervical Traction (Relieve Pain and Increase Flexibility)   Note: Each set 3-5 times, 3 sets | 10 minutes |
|  |  | 1. Soft Tissue Massage  - Scalene Muscles - Pectoralis Muscles - Levator Scapula Muscles - Upper Trapezius Muscles - Suboccipital Muscles - Occipital Muscles   Note: Each area approximately 2-3 minutes | First session: 10 minutes; subsequent sessions: 25 minutes |
| Phase 2  (4-6 weeks, 6 sessions, 60 minutes per session) | 1. Alleviate thoracic spine pain 2. Improve thoracic spine mobility | 1. Joint Mobilization Techniques (Matland, High-Velocity Low-Amplitude)  - Central PA Glide Mobilization of the Thoracic Spine (to improve flexion/extension) - Intervertebral Joint Mobilization (to improve lateral flexion) - Unilateral PA Mobilization of the Vertebrae (to enhance flexibility) - Multi-Vertebrae Rotational PA Mobilization (to enhance flexibility)   Note: 3-5 repetitions per set, 3 sets | 10 minutes |
|  |  | 1. Soft Tissue Massage  - Pectoral Muscles - Trapezius - Rhomboids - Splenius Capitis & Cervicis - Serratus Anterior - Thoracic Erector Spinae   Note: 3-5 repetitions per set, 3 sets | 25 minutes |
|  |  | 1. Physical Modalities  - Ultrasound Therapy - Compex Neuromuscular Electrical Stimulation   Note: Focusing on trigger points near thoracic spine muscles | 25 minutes |
| Phase 3  (7-8 weeks, 4 sessions, 60 minutes per session) | Enhance Proprioception | 1. Pain Management  - Physical Modalities: Shockwave Therapy, Compex Neuromuscular Electrical Stimulation, Ultrasound Therapy - Joint Mobilization Techniques - Soft Tissue Massage   Note: Therapist will customize treatment methods and target areas based on patient assessment | 20 minutes |
|  |  | 1. Static Stretching for Cervical and Shoulder Muscles  - Upper Trapezius - Sternocleidomastoid - Levator Scapulae - Scalene Muscles - Suboccipital Muscles - Rhomboids - Middle Trapezius - Pectoralis Major - Pectoralis Minor - Latissimus Dorsi | 40 minutes |

MPP protocol Note: Patient education is conducted procedurally during the initial treatment session, lasting approximately 20 minutes. The above techniques or movements are selected progressively as the patient's treatment advances and their capabilities are assessed.
